# Multiorgan resection with inferior vena cava reconstruction for hepatic alveolar echinococcosis: A case report and literature review

**DOI:** 10.1097/MD.0000000000003768

**Published:** 2016-06-10

**Authors:** Wei Li, Hong Wu

**Affiliations:** Department of Liver Surgery, West China Hospital of Sichuan University, Chengdu, China.

**Keywords:** alveolar echinococcosis, inferior vena cava, multiorgan resection

## Abstract

Alveolar echinococcosis (AE) is a life-threatening parasitic disease characterized by its tumor-like growth. Radical operation is deemed the curable method for AE treatment if R0-resection is achievable. We present a 26-year-old AE patient with AE lesions invading the right lobe of the liver, the inferior vena cava, inferior lobe of right lung, the right hemidiaphragm, and the right kidney. On the basis of precise preoperative and intraoperative evaluations, a radical surgery that removed the huge lesion en bloc was performed successfully with skillful surgical techniques. This patient had an uneventful postoperative recovery and a good prognosis. Multiorgan resection is justified and unavoidable in selected patients when AE lesions invade different organs and the main vascular structures.

## 1 Introduction

Echinococcosis granulosus (EG) and echinococcus multilocularis (EM) are 2 major medically significant parasites leading to hepatic echinococcosis. AE caused by the tumor-like growth of metacestodes of EM is a more malignant zoonosis than cystic echinococcosis (CE) resulting from EG infection in the liver.^[1]^ The association with dogs will lead to EM infection when dogs have eaten infected rodents such as foxes (which is often the definitive host of EM).^[2]^ Developing predominantly in the liver, metacestodes of EM can infiltrate or transfer to other organs such as the lung, kidney, and brain. In the liver, the larval mass proliferates by exogenous budding and the liver was damaged by direct erosion, mechanical compression, and toxic effect of EM, which may finally lead to liver failure and potentially life-threatening hepatobiliary complications, including jaundice, ascites, and gastroesophageal varices associated with severe cirrhosis.^[3]^ AE, occurred predominantly in the individuals aged 20 to 40, is characterized by a chronic course often with an initial asymptomatic incubation period of 5 to 15 years, and consequently only 35% of patients are eligible for partial hepatectomy, as most patients with AE are diagnosed at a later stage in our center.^[4]^

According to the expert consensus about the diagnosis and treatment of AE,^[1]^ the diagnosis of AE is based on detailed clinical presentation, epidemiological evidence, radiological examination, nucleic acid detection, and serologic tests. Ultrasound examination, computed tomography (CT), and magnetic resonance imaging (MRI) are the most commonly used techniques for pre-operative evaluation.^[5]^ Now, it is widely accepted that curative surgery for AE should meet the criteria of R0-resection just like the criteria for hepatocellular carcinoma. In principle, radical surgery should not be done unless R0-resection is achievable. Multiorgan involvement is also the surgical indication if the lesions can be removed totally.^[1]^. In this article, we present an end-stage AE patient whose right lobe of the liver, IVC, inferior lobe of right lung, the right hemidiaphragm, and the right kidney were infiltrated by the hepatic hydatid. And, we successfully removed the parasite-infiltrating organs with expanded polytetrafluoroethylene (ePTFE) replacing IVC and Dacron flap repairing diaphragmatic defect.

## 2 Case presentation

### 2.1 Preoperative evaluation and treatment

**Figure 1:**
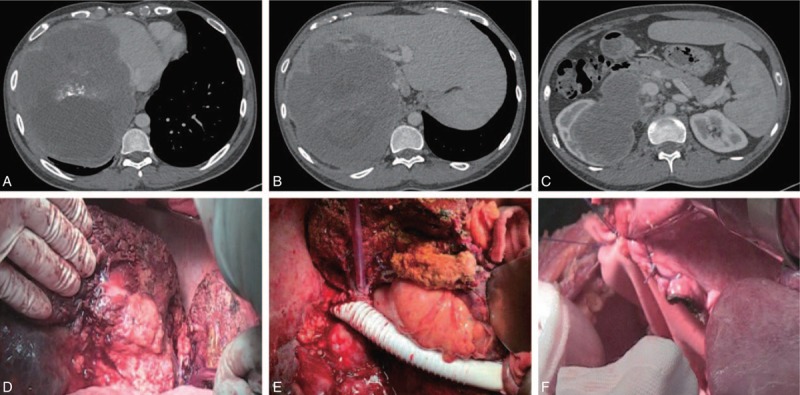
(A–C) Enhanced CT indicated a huge mass of 18 cm in diameter, which was in the right lobe of the liver with IVC and the right kidney invasion. The black arrays: IVC. The white array: portal vein. (D) The parasitic lesion in the right liver. (E) Repair the diaphragm with Dacron patches. (F) Reconstruct the IVC by an end-to-end anastomosis with an ePTFE graft, the array: ePTFE graft.

A 26-year-old man from the pastoral area of west China, presenting with slightly abdominal pain, was transferred to our center. Two years ago, a lesion was found in the liver of the patient by contrast-enhanced CT in the local hospital, but this patient had refused any recommended treatment because of no obvious symptoms. In our hospital, the MRI and CT showed that a huge cystosolid lesion about 15.7 × 19 cm was in the right lobe of the irregularly shaped and volume-increased liver. The IVC, right and middle hepatic vein, parts of portal triad, right kidney, and the right part of the diaphragm were compressed and invaded (Fig. 1A, B, and C). In addition, the intrahepatic bile duct was slightly dilated due to the obstruction of the lesion. The volume of the residual healthy liver was 450 mL. No distant metastasis was found by the CT scan of the brain and the left lung. The laboratory results were as follows: hemoglobin: 112 g/L, total bilirubin: 21 mmol/L, direct bilirubin: 7.6 mmol/L, aspartate aminotransferase: 31 IU/L, alanine aminotransferase: 32 IU/L, albumin: 35 g/L, prothrombin time: 14.2 seconds, and international normalized ratio: 1.27. Serologic tests demonstrated that the immunoglobulin G (IgG) aiming at the echinococcus was positive. In addition, the quantification of hepatitis B and E surface antigen is positive and hepatitis B virus (HBV)-DNA real-time fluorescence detection was 5,390,000 IU/mL. After multidisciplinary discussion, with nearly 2 months of antiviral therapy, the patient's HBV-DNA recovered to a normal level. We finally decided to carry out this tough procedure for the young patient with the approval of his family members. The surgical procedure and this report have been approved by the ethics committee of West China Hospital of Sichuan University.

### 2.2 Surgical technique and postoperative management

The surgery was performed in cooperation with the liver transplantation center, vascular center, thoracic surgery center, and urinary surgery center. Thoracoabdominal inverse L-shaped incision was chosen for this complicated procedure to provide excellent exposure of the thoracic, abdominal cavity. After carefully surgical exploration, intraoperative ultrasonography was done, which found an indurated huge lump with a marked upward extension involving the inferior lobe of right lung and the right hemidiaphragm, and with a prominent downward extension involving the right kidney. The IVC was infiltrated and the inner diameter of it was less than 0.5 cm. The right hepatic vein did not appear and the middle hepatic vein was only 0.4 cm in diameter. The confluence of the 2 hepatic veins was also involved by the lesion. The left lateral lobe of the liver showed a compensatory increase and no lesions were found within it.

After cholecystectomy, the portal structures were carefully isolated and the hepatic pedicles of the right and left medial lobe, including the hepatic artery, portal vein, and bile duct, were ligated. Thereafter, by Kocher maneuver, the IVC was detached from the retroperitoneum and secured 0.5 cm below the level of the right renal vein. Then, the right renal artery, vein, and ureter were sutured and ligated for the right kidney was extensively destroyed. The suprahepatic IVC was exposed after resection of the inferior lobe of right lung and the right hemidiaphragm. Then, the right trisectionectomy (Fig. 1D) was carried out with intermittent Pringle maneuver. When the liver parenchymal transection reached to the left hepatic vein, the portal structures, infrahepatic vena cava 1 cm below the right renal vein, and suprahepatic vena cava just below the confluence into the atrium were clamped. After cutting off the infrahepatic vena cava, the IVC was separated from the retroperitoneum upward and to the left hepatic vein confluence. With these procedures, the IVC was completely mobilized, and the vascular clamp on the suprahepatic vena cava was moved to the level just below the left hepatic vein confluence. Simultaneously, the portal inflow occlusion was released allowing continued perfusion of the liver with portal and arterial circulation. After that, the right trisegmentectomy specimen, the right kidney, and the involved IVC were removed en bloc. The prepared artificial blood vessel was then sewn end to end to the supra- and infrahepatic vena cava (Fig. 1E). After the clamps were released, there was excellent blood flow through the graft. Finally, the right hemidiaphragm was repaired using the Dacron patches (Fig. 1F).

**Figure 2:**
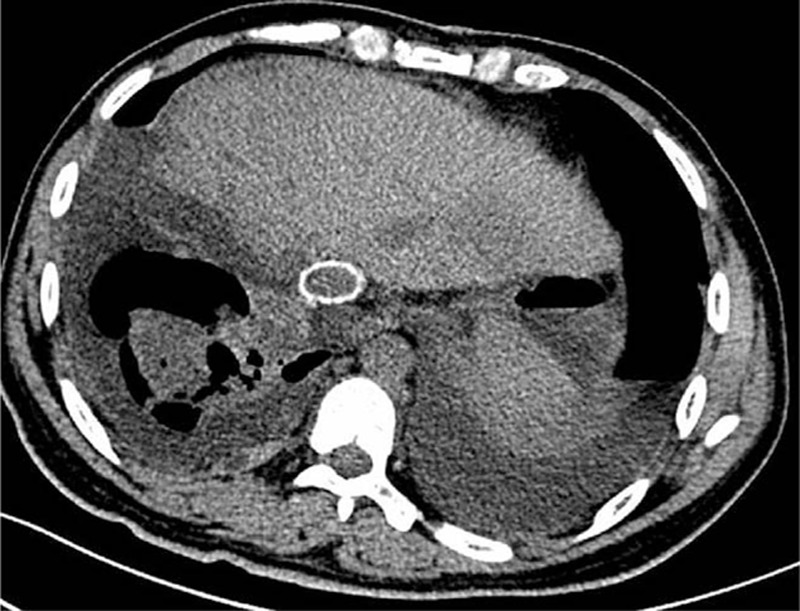
The CT scan at 15 days after the surgery indicated a patent IVC with no obstruction.

**Figure 3:**
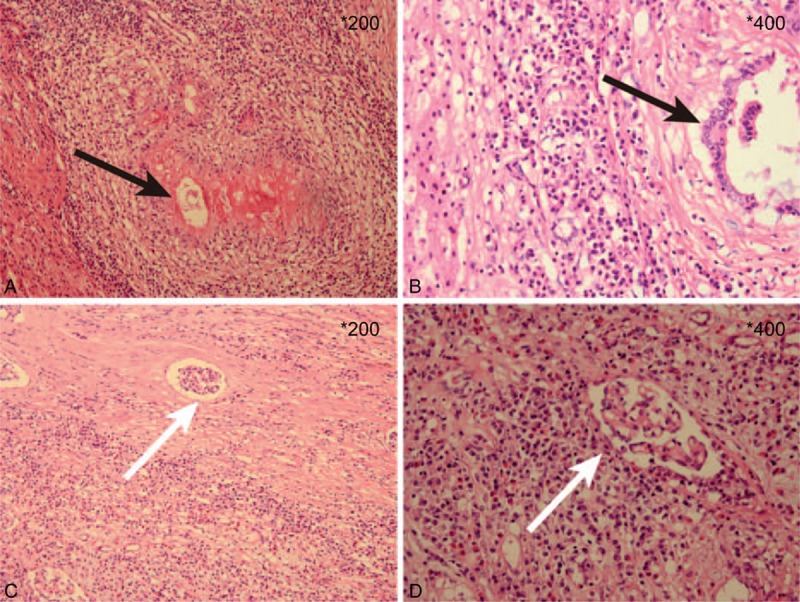
Hematoxylin and eosin stain of paraffin sections. (A, B) Histopathology of liver lesion (magnification of A: 200, B: 400). (C, D) Histopathology of the kidney lesion (magnification of C: 200, D: 400). The arrows: AE.

The surgical procedures lasted for nearly 17 hours and the patient's hemodynamics remained stable. The patient was transfused with 12 units of erythrocyte suspension and 2000 mL of fresh-frozen plasma, and the blood loss was about 2500 mL. The postoperative management included antibiotic therapy, anti-coagulative therapy (continuous intravenous anticoagulant treatment with heparin was adopted until postoperative day 8 and then the patient was given warfarin for 3 months), and anti-HBV therapy, etc. No severe postoperative complications occurred (grade II according to the modified Clavien classification system)^[6]^ and the liver function returned to norm on day 8 postoperatively. The CT imaging on postoperative day 15 demonstrated a patent IVC graft (Fig. 2). He was discharged from the hospital on postoperative day 18 and is doing well at home currently, back at work, and without recurrence 6 months after the operation. And, the final pathology report was consistent with AE and the incisal margin was clear (Fig. 3).

## 3 Discussion

According to the latest recommendations of the WHO-Informal Working Group on Echinococcosis (WHO-IWGE) for the management of human AE,^[1]^ radical surgery (R0-resecton) is the primary goal, as AE is characterized by tumor-like infiltrative growth. Any palliative operations for reducing the parasitic burden do not appear currently to acquire advantages over antiparasitic drug treatment. However, most patients are in later stages and radical surgery cannot be performed. On the basis of the WHO classification,^[1]^ our case was in P4N1M0 phase. The preoperative evaluations, including cerebral, thoracic, and abdominal imaging examinations, demonstrated no distant metastasis and it was most possible a direct infiltration to the perihepatic regions, including the right lobe of the liver, IVC, inferior lobe of right lung, right hemidiaphragm, and right kidney. Lesions not confined to the liver are not a contraindication to surgery once curative procedures meet the criteria of R0-resection.^[1,7]^ In this case, the lesions confining to 1 surgery field, we performed radical resection after improved preoperative examination and intraoperative assessment by experienced surgeons. However, if a patient had distant metastasis such as in the brain, would we perform a radical operation?^[8]^ Therapeutic options for patients with AE at this stage need further demonstration. Whether the patient should be managed by surgery or alternative drug therapy remains controversial.

For this patient, we removed the IVC and reconstructed it with an artificial graft because it was invaded by AE and cannot be separated, thus IVC resection may be necessary to achieve R0 resection. However, in our experience, there are some situations when IVC replacement is not necessary: First, parasite lesions can be totally separated from IVC without creating a caval defect. Second, our rule of thumb has been: if less than 50% of the IVC circumference is flattened, we close the IVC by a simple continuous transversely suture or using patches including the autogenous vein (such as the great saphenous vein) patch graft and ePTFE patch graft. Third, if venous collaterals such as ascending lumbar veins, hemiazygos vein, and azygos vein are dilated and compensate portal hypertension and caval flow effectively, we will not consider IVC replacement even more than 50% of the circumference of the IVC wall was involved. There is controversy about whether IVC resection is justified for hepatic echinococcosis especially CE.^[9]^ In our experience, for CE, if a patient has severe symptoms and life-threatening complications for caval obstruction and portal hypertension (collateral circulations cannot compensate) and the lesions cannot be separated from IVC, then the combined resection of the liver and IVC may be considered. As for AE, we believe that radical surgical treatment (R0 resection) is one of the most important surgical indications due to the tumor-like growth of the EM larvae in the liver. In this case, it is feasible to replace the IVC with prosthetic material and it can produce long-term patency.

In order to achieve R0 resection, for this patient, the right hepatic trisegmentectomy and right nephrectomy were performed after precise preoperative and intraoperative evaluations. Radioactive renogram of the uninvolved left kidney and other renal function tests showed that the right kidney can be removed safely. The preoperative CT indicated that the volume of the enlarged left lateral lobe was 450 mL. The ICG retention at 15 minutes was less than 10%. And, this patient had no cirrhosis for parasite infection. Intraoperative ultrasound showed that the middle and left hepatic veins were invaded by AE and right hepatic trisegmentectomy was necessary for obtaining parasite-free margins.

During the operation, we adopted 2-step hepatic vascular exclusion in order to shorten warm ischemia time of the liver (40 minutes in this patient) and alleviate ischemia-reperfusion injury of the remnant liver. Azoulay et al^[10]^ introduced 2-step hepatic vascular occlusion in detail. Given the fact that TVE was safe for up to 60 minutes in many studies,^[11,12]^ hypothermic liver perfusion was not utilized during the surgery. The patient's hemodynamics remained nearly stable after clamping the infra- and suprahepatic IVC; thus, venovenous bypass was not used in this patient.

In conclusion, multiorgan infiltration is not a surgical contraindication for AE. Given the lack of alternative curative approaches, a radical surgery with complete removal of the parasitic lesions is the only way to achieve radical treatment. Awareness and application of surgical techniques, which permit removal of lesions with vascular invasion, may expand the indications for surgical excision in selected cases.
